# Piezo‐Phototronic Effect Enhanced Flexible Solar Cells Based on n‐ZnO/p‐SnS Core–Shell Nanowire Array

**DOI:** 10.1002/advs.201600185

**Published:** 2016-07-07

**Authors:** Laipan Zhu, Longfei Wang, Fei Xue, Libo Chen, Jianqiang Fu, Xiaolong Feng, Tianfeng Li, Zhong Lin Wang

**Affiliations:** ^1^ Beijing Institute of Nanoenergy and NanosystemsChinese Academy of SciencesNational Center for Nanoscienceand Technology (NCNST)Beijing100083P. R. China; ^2^ Microsystems and Terahertz Research CenterChina Academy of Engineering PhysicsChengduSichuan610200China; ^3^ School of Material Science and EngineeringGeorgia Institute of TechnologyAtlantaGA30332USA

**Keywords:** core–shell nanowire arrays, flexible solar cells, n‐ZnO/p‐SnS, piezo‐phototronic effect, piezopotential

## Abstract

The piezo‐phototronic effect is about the enhanced separation, transport, and recombination of the photogenerated carriers using the piezoelectric polarization charges present in piezoelectric‐semiconductor materials. Here, it is presented that the piezo‐phototronic effect can be effectively applied to improve the relative conversion efficiency of a flexible solar cell based on n‐ZnO/p‐SnS core–shell nanowire array for 37.3% under a moderate vertical pressure. The performance of the solar cell can be effectively enhanced by a gentle bending of the device, showing its potential for application in curly geometries. This study not only adds further understanding about the concept of increasing solar energy conversion efficiency via piezo‐phototronic effect, but also demonstrates the great potential of piezo‐phototronic effect in the application of large‐scale, flexible, and lightweight nanowire array solar cells.

## 1 Introduction

With the threat of an upcoming energy shortage and deterioration of the environment, great efforts are being devoted to develop renewable and green energy resources. Photovoltaic that converts sunlight to electricity is one of the promising technologies to solve the energy problem that our society faces in the near future. Because semiconductor nanowires (NWs) have a lot of advantages such as large surface‐to‐volume ratio, better charge collection,^1^ and the possibility of enhanced absorption through light trapping,^2^ so that NWs‐based photovoltaics (PV) have been the subject of research for enhancing the energy conversion efficiency.^3^ On the other hand, nanowires will cause large surface and interface recombination, which could be overcome by surface passivation and epitaxial growth of p‐n junctions.^4^, ^5^ The core–shell geometry of NWs is proposed to be able to enhance the efficiency of charge collection by shortening the paths traveled by minority carriers, increasing the optical quality of the material. Furthermore, the flexible power devices exhibit several advantages over established technologies.^6^, ^7^ Thus, NWs‐based PV will have great application in flexible power source compared to bulk materials. However, a large source of loss in this type of device is nonradiative recombination of the photogenerated carriers.^8^ Therefore, new strategies are necessary to reduce this recombination and significantly increase device efficiency.

The fundamental principle of piezotronics and piezo‐phototronics was introduced by Wang and co‐workers in 2007 and 2010, respectively.^9^, ^10^, ^11^, ^12^ Due to the polarization of ions in a crystal that has noncentral symmetry in materials such as the wurtzite structured ZnO, GaN, and InN, a piezopotential is created in the crystal by applying a stress. Piezotronics is about the devices fabricated using the piezopotential as a “gate” voltage to tune/control charge carrier transport at a contact or junction. Piezotronics are likely to have important applications in sensors, human‐silicon technology interfacing, micro‐electromechanical systems (MEMS), nanorobotics, electronic skin, and active flexible electronics.^11^, ^12^, ^13^ The piezo‐phototronic effect is to use the inner‐crystal piezo‐charges generated piezopotential to control the carrier generation, transport, separation and/or recombination for improving the performance of optoelectronic devices, such as photon detector, solar cell, and light‐emitting diode (LED).^10^, ^11^, ^12^, ^13^, ^14^ In these piezoelectric semiconductor nanowires, because of their noncentrally symmetric crystal structure, piezoelectric polarization charges can be created at the end of each nanowire by applying a strain, pressure or force. Indeed, although there have been many great studies on flexible solar cells based on single piezoelectric nanowire,^5^, ^15^, ^16^ it is almost unable to be integrated for large‐scale PV devices to get more output power.

In this paper, we report the enhanced performance of a large‐scale flexible photovoltaic device based on n‐ZnO/p‐SnS core–shell nanowire (NW) array using the piezo‐phototronic effect for the first time. SnS (with an electron affinity of 4.0 eV and a high optical absorption coefficient of 10^4^ cm^−1^) has a direct optical energy band gap (*E*_g_) of 1.3 eV, close to the optimum value required for efficient light absorption,^17^, ^18^ and it further shows an intrinsic p‐type conductivity with carrier concentrations in the range of 10^15^–10^18^ cm^−3^ due to the easy formation of acceptor‐like intrinsic Sn vacancy defects,^18^, ^19^ which make it a good shell material for core–shell solar cells (Figure S1, Supporting Information). The piezopotential of ZnO nanowire array is used as driving force to promote effectively the separation and transport of photogenerated carriers via engineering the band structure of the ZnO/SnS heterojunction. This study shows the potential of piezo‐phototronics for high performance large scale flexible solar cell applications.

## 2 Results and Discussion

**Figure 1:**
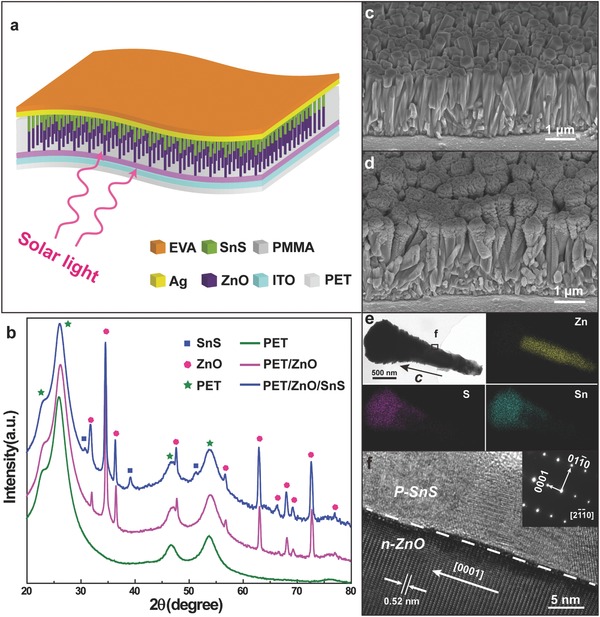
Structural characterization of n‐ZnO and n‐ZnO/p‐SnS core‐shell NWs. a) The schematic diagram of the as‐synthesized flexible solar cell based on n‐ZnO/p‐SnS core–shell NW array. b) X‐ray diffraction (XRD) spectra of the PET (the green line), PET/ZnO (the pink line), and PET/ZnO/SnS (the blue line). The peaks in the blue line marked with green pentastars, pink dots, and blue squares belong to PET substrate, ZnO cores, and SnS shells, respectively. c) Side view of SEM image of the morphology of the as‐synthesized ZnO NW array. d) Side view of SEM image of the morphology of the n‐ZnO/p‐SnS core–shell NW array. e) Low‐magnified TEM image and EDX mapping of the n‐ZnO/p‐SnS core–shell NW. f) HRTEM image of the n‐ZnO/p‐SnS core–shell NW, the inset in the upper left corner denoting the SAED pattern of the ZnO NW.

The PV device is based on an array of n‐ZnO/p‐SnS core–shell nanowires grown on an indium tin oxide (ITO) coated polyethylene terephthalate (PET) substrate, with the *c*‐axis of ZnO pointing upward from the substrate (**Figure** **1**a and Figure S2, Supporting Information), thereby forming an array of p‐n heterojunction solar cells. Figure 1b illustrates X‐ray diffraction (XRD) spectra corresponding to PET, PET/ZnO, and PET/ZnO/SnS, respectively. The n‐ZnO NWs are synthesized via a hydrothermal method, which give high quality and orientation‐uniform NWs (Figure 1c and Figure S3, Supporting Information). The morphology of ZnO/SnS core–shell nanowire array on ITO coated flexible PET substrate is also presented (Figure 1d and Figure S4, Supporting Information). The low‐magnification transmission electron microscopy (TEM) image and the energy‐dispersive X‐ray (EDX) mapping of a ZnO/SnS core–shell nanowire (Figure 1e) demonstrate that Sn and S are distributed at the shell (the thickness of the shell is about 0–500 nm) while the Zn located at the core. A high‐resolution transmission electron microscopy (HRTEM) image and the corresponding selected area electron diffraction (SAED) pattern taken from the edge of ZnO NW are presented (see Figure 1f), indicating that the ZnO core is structurally uniform and single crystalline with length direction along the *c*‐axis and that the SnS shell is polycrystalline.

**Figure 2:**
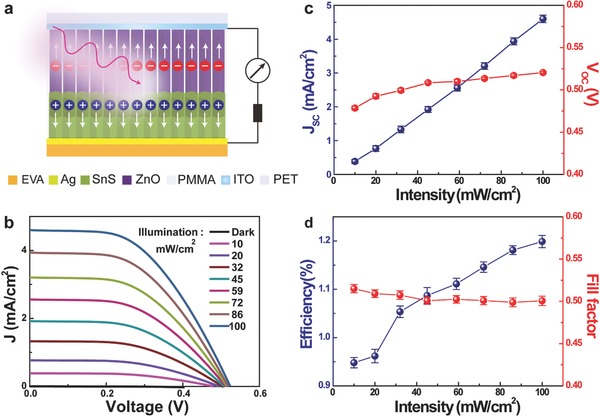
Performance of the flexible solar cell based on n‐ZnO/p‐SnS core–shell NW array at different illumination intensities. a) The schematic cross section diagram of the solar cell. b) *J*–*V* characteristics of the solar cell at different illumination intensities. c) The illumination intensity dependence of the short‐circuit current density (*J*_SC_) and the open‐circuit voltage (*V*_OC_). d) The illumination intensity dependence of the solar energy conversion efficiency (*η*) and the fill factor (FF).

The schematic cross section diagram of the as‐synthesized flexible ZnO/SnS core–shell NW array solar cell is shown in **Figure** **2**a with the sun light incidenting from the PET substrate. Figure 2b demonstrates the *J*–*V* characteristics of a typical cell under different illumination intensities (*P*), ranging from 10 to 100 mW cm^−2^. The dependency of the performance characteristics on the illumination intensity is shown in Figure 2c,d. Specifically, an efficiency (*η*) of ≈1.2% is obtained with an open‐circuit voltage *V*_OC_ ≈ 0.52 V, short‐circuit current density *J*_SC_ ≈ 4.6 mA cm^−2^, and fill factor FF ≈ 0.43 under AM 1.5G illumination. On the one hand, *J*_SC_ exhibits a near‐linear dependency on the intensity, which is due to that the photocurrent in this regime is proportional to the photon flux or the carrier generation rate with a constant minority carrier lifetime. On the other hand, *V*_OC_ increases only slightly from 0.47 to 0.52 V with the increase of intensity, which can be attributed to a slight thermal heating of the device.^20^, ^21^ The fill factor FF decreases slightly with illumination intensity. As the solar energy conversion efficiency can be expressed as *η* =*V*_OC_ ×*J*_SC_ × FF/*P*, the calculated *η* is enhanced with the increase of illumination intensity, as shown in Figure 2d, which indicates that the carrier collection efficiency is higher for the solar cell under high illumination intensity. In other words, although there are a large number of photogenerated electron–hole pairs under high illumination intensity, it can be sufficiently separated at the interface. The performance of such core–shell ZnO/SnS NW array solar cell can be enhanced by optimizing the structure (such as adding antireflective surface coating), impurity doping, metal–semiconductor contact, etc.

**Figure 3:**
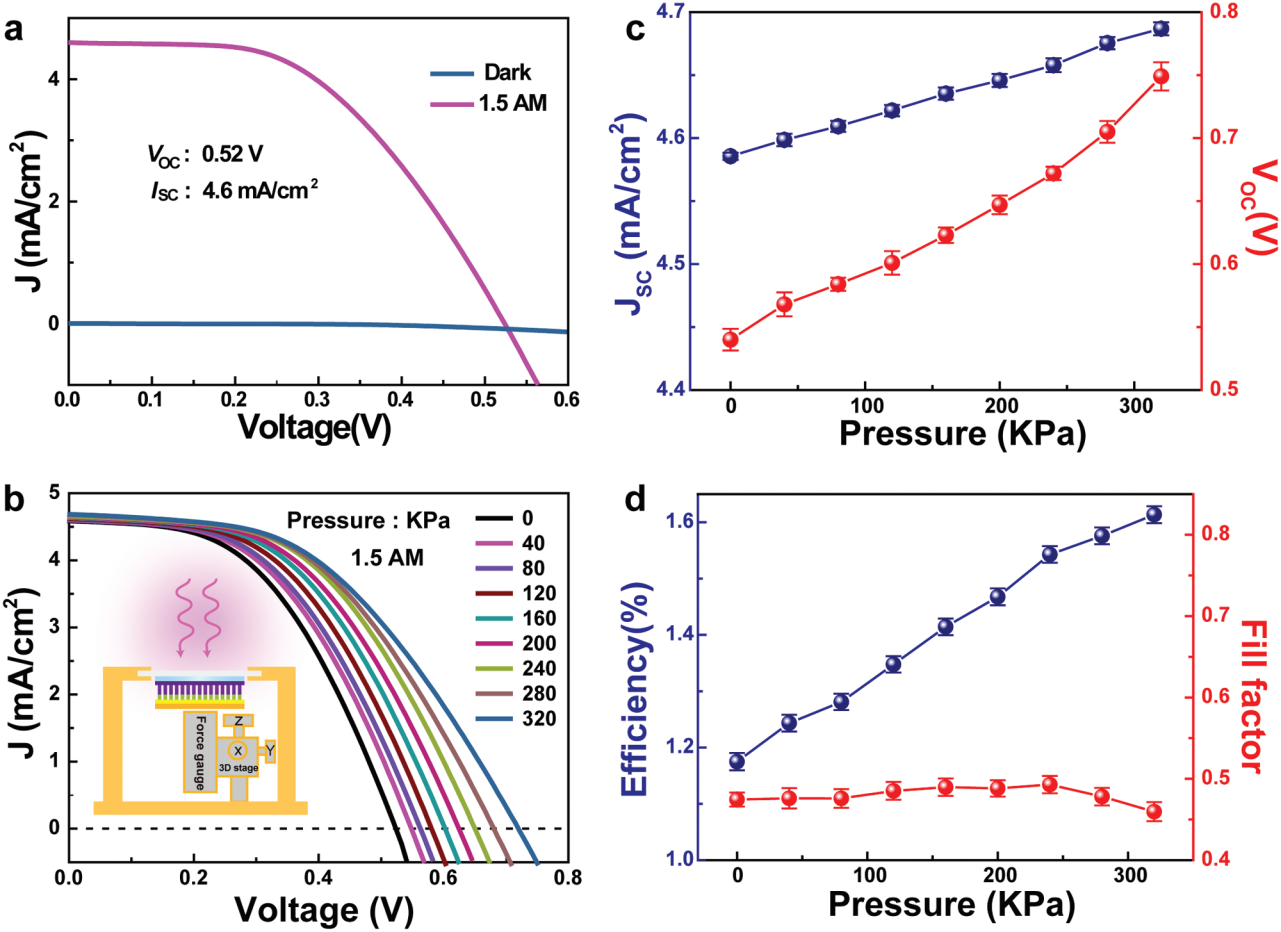
Performance of the flexible solar cell based on n‐ZnO/p‐SnS core–shell NW array at different vertical pressures. a) *J*–*V* characteristics of the solar cell in dark or under 1 sun (AM 1.5G) illumination. b) *J*–*V* characteristics of the core–shell NW array at AM 1.5G illumination and different external vertical pressures. c) Vertical pressure dependence of the short‐circuit current density and the open‐circuit voltage. d) Vertical pressure dependence of the solar energy conversion efficiency and the fill factor.

To investigate the effects of the piezopotential on the performance of such core–shell ZnO/SnS NW array solar cell, *J*–*V* characteristics of the solar cell under a variety of vertical pressures (40–320 KPa on the 5 mm × 5 mm EVA foam) are measured under AM 1.5G illumination, as shown in **Figure** **3**b (Figure 3a is a contrast for Figure 3b). As the substrate is a flexible PET, the PV device is fixed first on a high transparent glass in order to bear the vertical pressure. The vertical pressure dependence of the performance of the core–shell ZnO/SnS NW array solar cell is shown in Figure 3c,d, which implies that the performance of such PV device is enhanced with the increase of applied vertical pressure. Specifically, the *J*_SC_ and the *V*_OC_ under different vertical pressures are extracted and plotted in Figure 3c. It can be found that the *J*_SC_ enlarges from 4.58 to 4.69 mA cm^−2^ for only 2.4%, while the *V*_OC_ increases from 0.54 to 0.75 V for up to 39% fluctuation. The fill factor FF is about 0.48, which is almost invariable with the change of vertical pressure. As a result, the solar energy conversion efficiency is significantly increased by 37.3% when a 320 KPa pressure is applied. Such enhanced performance of the solar cell under a vertical pressure is suggested due to the effective increase of the built‐in electric field at the heterojunction interface between ZnO and SnS caused by piezoelectric polarization charges,^10^, ^11^, ^22^ which will be discussed below.

**Figure 4:**
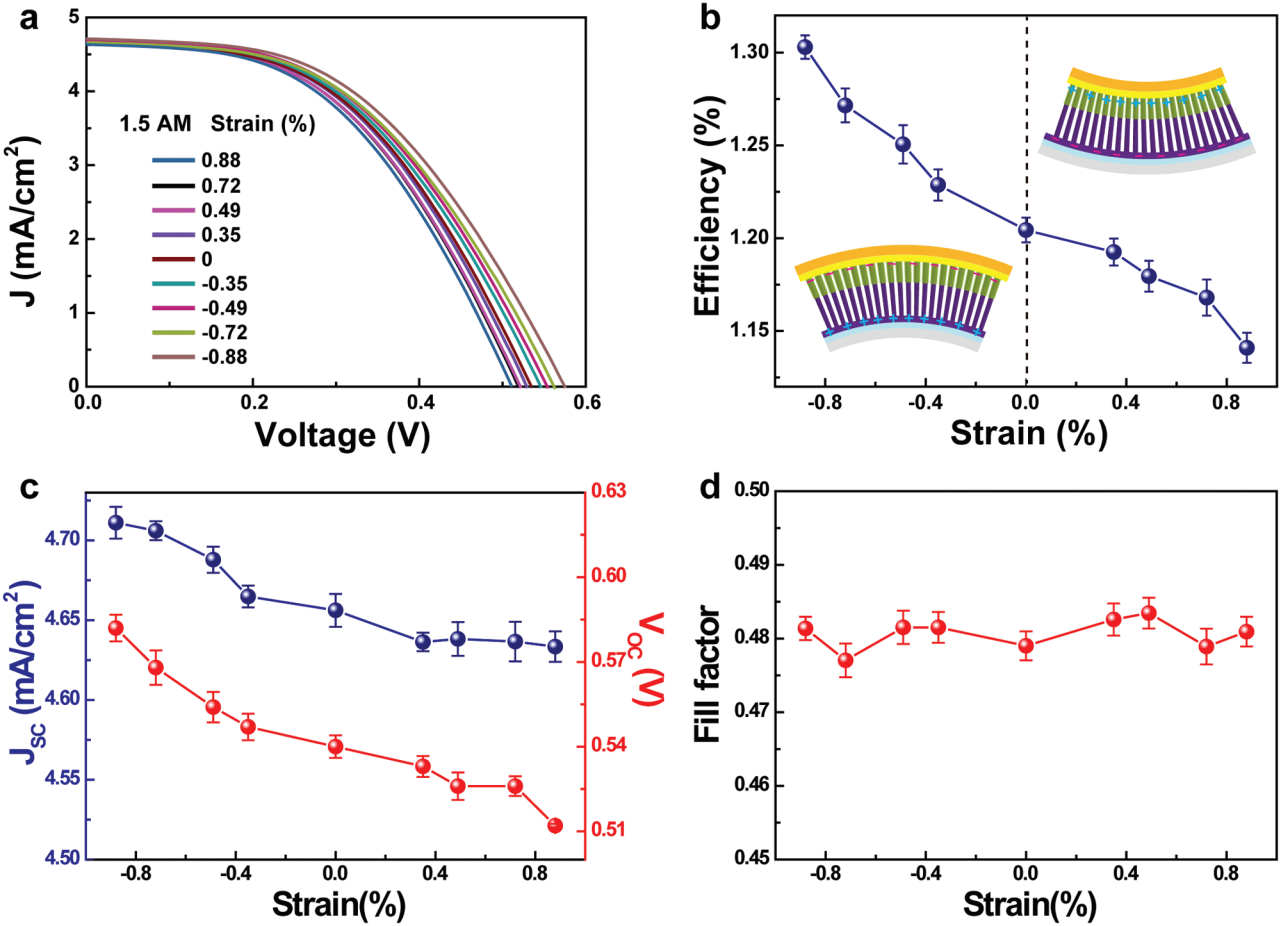
Performance of the flexible solar cell based on n‐ZnO/p‐SnS core‐shell NW array at different bending strains. a) *J*–*V* characteristics of the solar cell at different bending strains and under AM 1.5G illumination, where the strain denotes the change of the length of the bottom of PET substrate. b) Bending strain dependence of the solar energy conversion efficiency. c) Bending strain dependence the short‐circuit current density and the open‐circuit voltage. d) Bending strain dependence of the fill factor.

Mechanically flexible solar cells are of particular interest for a number of practical applications.^6^ In this regard, we studied the *J*–*V* characteristics of the core–shell ZnO/SnS NW array solar cell in different bending strains (−0.88%–0.88%) under AM 1.5G illumination, as shown in **Figure** **4**a. Here the bending strain is defined as compressive or tensile strain of the bottom of the PET substrate (i.e., the surface of first contacting the sun light, see the insets of Figure 4b) when bending downward or upward the PET substrate (with 250 μm in thickness, regardless of the thickness of the ITO electrode). What calls for special attention is that the core–shell ZnO/SnS NW array will suffer from a compressive strain when applying a compressive strain on the bottom of PET. Similarly, the NW array will suffer from a tensile strain when applying a tensile strain on the bottom of PET. As illustrated in the two insets of Figure 4b, the distribution of the piezoelectric polarization charges is reversed for the two conditions. The performance of this PV device is enhanced when the NW array is subjected to a compressive strain (downward‐bending strain) while decreased when subjected to a tensile strain (upward‐bending strain).^22^, ^23^ And the *J*_SC_, the *V*_OC_, and the FF under different strains are extracted and plotted in Figure 4c,d. With the strain changing from a compressive strain of −0.88% to a tensile strain of 0.88%, it can be found that the *J*_SC_ decreases from 4.71 to 4.63 mA cm^−2^, and the *V*_OC_ decreases from 0.58 to 0.51 V, while the FF stabilizes around 0.48. As a result, the conversion efficiency *η* increases from 1.2% to 1.3% for nearly 8.3% with application of a variable compressive strain from 0% to −0.88%, while *η* decreases from 1.2% to 1.14% about 5% with application of a variable tensile strain from 0% to 0.88% (see Figure 4b). That is to say, the performance of the PV device can be effectively enhanced by certain bending of the device, showing its potential for application in curly geometries. These changing tendencies are in agreement with the result expected from the piezo‐phototronic model, to be presented.

**Figure 5:**
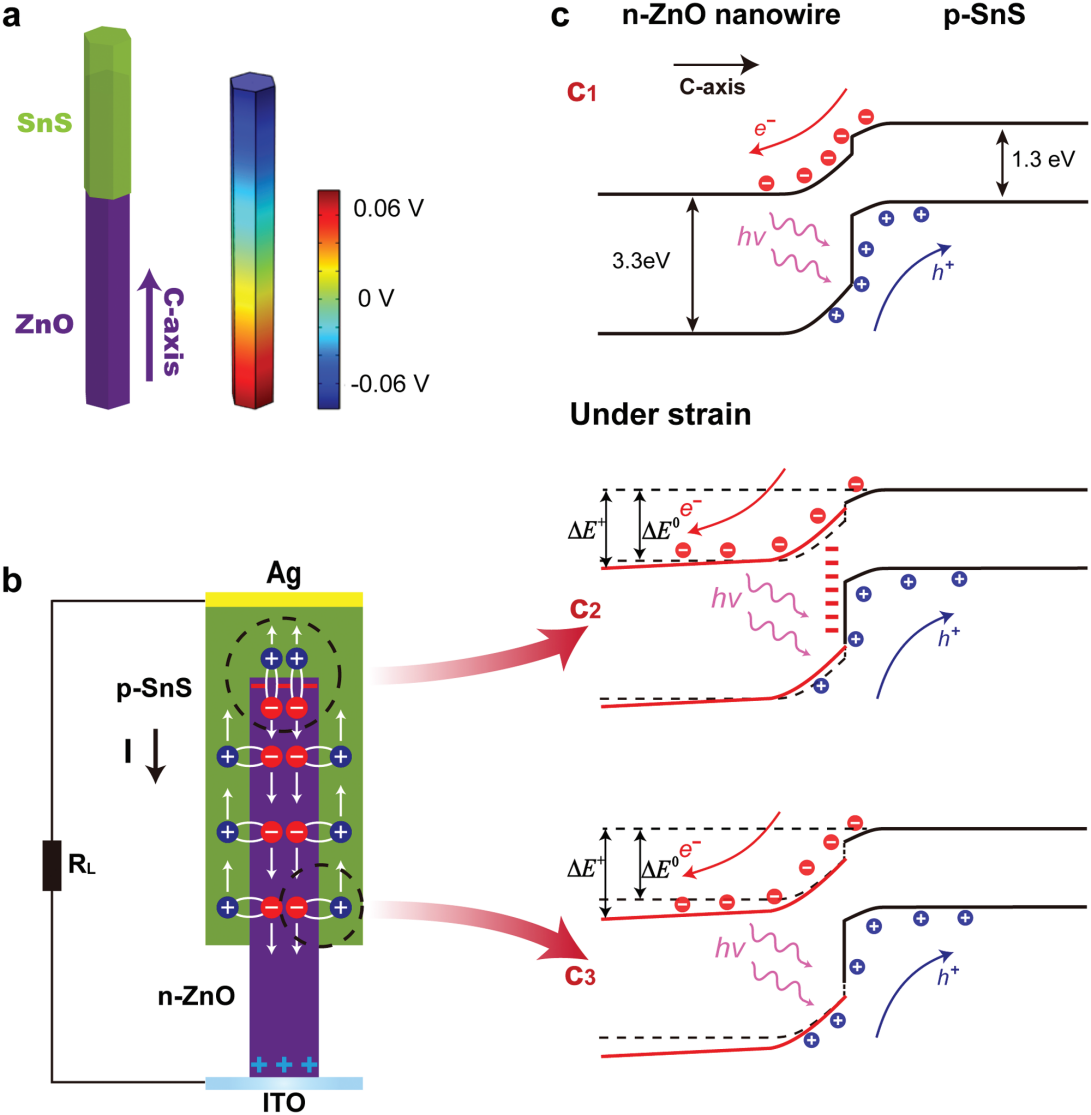
Proposed mechanism for enhanced solar energy conversion efficiency by the piezo‐phototronic effect. a) Numerically calculated piezopotential distribution simulated by a finite‐element analysis method (COMSOL), where the diameter, the length, and the applied pressure are used to be 300 nm, 3 μm, and 500 KPa, respectively. b) Schematic diagram exhibiting the separation and transport of the photoinduced electron–hole pairs in an n‐ZnO/p‐SnS core–shell NW under light illumination. c) Energy band diagrams of the n‐ZnO/p‐SnS NW array solar cell before (c_1_) and after (c_2_ and c_3_) applying a compressive force on the PV device.

Since the p‐SnS shell is polycrystalline so that it does not have a measurable piezoelectric effect, our discussions mainly focus on the piezoelectric effect from the ZnO core. ZnO has a noncentral symmetric wurzite structure in which the cations and anions are tetrahedrally coordinated. A strain on the basic unit results in a polarization of the cations and anions, which is the cause of the piezopotential inside the crystal. The piezo‐phototronic effect enhanced performance of ZnO/SnS core–shell NW array solar cell under pressure/strain can be qualitatively explained through the schematic structure, numerically calculated piezopotential distribution, schematic diagram exhibiting the separation and transfer of carriers, and the corresponding energy band diagram under the influence of the inner piezopotential, as shown in **Figure** **5**. The numerically calculated piezopotential distribution in Figure 5a is simulated by a finite‐element analysis method (COMSOL), where the diameter, the length, and the applied pressure are 300 nm, 3 μm, and 500 KPa, respectively. It should be pointed out that the actual pressure applied on the core–shell NW array is much larger than that on the PV device, because of the interval in core–shell NW array.

On the one hand, the local negative charges induced by compressive strain at the ZnO/SnS top interface will lift up the conduction and valence bands of ZnO, as denoted in Figure 5c_2_, resulting in an increase of the barrier height at the heterojunction interface. This is equivalent to an increase in the depletion width and internal field, which will make the photoinduced electron–hole pairs easier to be separated especially for a direct band‐to‐band transition in ZnO NW cores (but almost no enhancement for the separation of electron–hole pairs in SnS shells) and thus decrease the possibility of recombination. Furthermore, the transport of the separated electrons will be further driven by the piezopotential in ZnO NW electron transport layer. On the other hand, although the separation of the photoinduced electron–hole pairs are not enhanced at the ZnO/SnS side interface as there is no piezoelectric charge, the transport of the separated carriers (especially electrons) can be effectively enhanced due to the piezopotential in ZnO, as denoted in Figure 5c_3_. Therefore, the output of photoinduced current *I*_L_ is increased when the device is compressively strained due to the above two mechanisms. The effective current *I* providing to a load resistor of the solar cell can be written as *I* = *I*_L_ −*I*_F_, where *I*_F_ is the positive current of p‐n heterojunction induced by the positive photovoltage.^21^ Under the condition of short‐circuit, the positive current *I*_F_ is equal to 0, so the effective current *I* is actually equal to the short‐circuit current *I*_SC_. In other words, the short‐circuit current *I*_SC_ is equal to the light induced current *I*_L_. Thus, *I*_SC_ (or *J*_SC_) is increased when the device is compressively strained,^22^, ^23^ as shown in Figures 3c and 4c. As a p‐n heterojunction solar cell, the fabricated PV device can be described by a Shockley equivalent circuit.^15^, ^21^, ^24^ The open‐circuit voltage *V*_OC_ and the reverse saturation current *I*_0_ can be expressed as

(1) $$V_{\mathrm{OC}}=\frac{k_{0}T}{e}\ln\left(\frac{I_{\mathrm{SC}}}{I_{0}}\right)$$

(2) $$I_{0}=I_{00}\exp\left(-\frac{\Delta E}{rk_{0}T}\right)$$

where *I*_SC_ is the short‐circuit current, *I*_00_ is a prefactor, *r* is an ideality factor, and Δ*E* is the energy band difference between the conduction band of a n‐type inorganic material and the conduction band of a p‐type inorganic material.^15^ By combining Equations (1) and (2), the open‐circuit voltage *V*_OC_ can be further expressed by

(3) $$eV_{\mathrm{OC}}=\frac{1}{r}\Delta E+k_{0}T\ln\left(\frac{I_{\mathrm{SC}}}{I_{00}}\right)$$

which indicates that the open‐circuit voltage *V*_OC_ is associated with both the energy band difference Δ*E* and the short‐circuit current *I*_SC_. Δ*E* will be increased with the increase of compressive strain due to the tilt of energy band in ZnO NW (see Figure 5c). As the Δ*E* and the *I*_SC_ are all increased when the device is compressively strained, the *V*_OC_ is then increased, as shown in Figures 3c and 4c. This is the basic mechanism of how does the piezo‐phototronic effect enhance the conversion efficiency of the core–shell solar cell. However, when the NW array is subjected to a tensile strain (i.e., an upward‐bending strain), the piezopotential distribution is reversed compared to that described for compressive strain. Eventually, the short circuit current *I*_SC_ (or the short‐circuit current density *J*_SC_), the open‐circuit voltage *V*_OC_, and the conversion efficiency *η* of the core–shell solar cell are all decreased compared to each corresponding value under zero strain,^22^, ^23^ as shown in Figure 4b,c.

## 3 Conclusion

We report that the piezo‐phototronic effect can be effectively applied to improve the relative conversion efficiency of a flexible solar cell based on n‐ZnO/p‐SnS core–shell nanowire array for 37.3% under a moderate vertical pressure of 320 KPa. And the performance of this PV device is enhanced when the NW array is subjected to a compressive (downward‐bending) strain while decreased when subjected to a tensile (upward‐bending) strain. Our study not only adds further understanding about the concept of increasing solar energy conversion efficiency via piezo‐phototronic effect, but also demonstrates great potential of piezo‐phototronic effect in the applications of large‐scale, flexible, and lightweight solar cells.

## 4 Experimental Section

First, thin layers of ITO transparent top electrode (Ar, 100 W, 40 min) and ZnO seed layer (Ar, 100 W, 15 min) were deposited in turn on PET substrate by radio frequency (RF) magnetron sputtering at room temperature. Then, the coated PET substrate was then placed into a mixed nutrient solutions (100 mm L^−1^ Zn(NO_3_)_2_ and 100 mm L^−1^ HMTA) at 80 °C for 4 h for ZnO NWs growth. SnS shells were then deposited on the exposed ZnO nanowire array by RF magnetron sputtering at 110 °C. For avoiding short‐circuit of the device and improving mechanical property of nanowires, the as synthesized ZnO nanowire array was then spin‐coated by PMMA (2 μm) followed by Ar plasma etching for 5 min. Then, the bottom electrode (Ag) was deposited by direct current (DC) magnetron sputtering at room temperature. Finally, a 5 mm × 5 mm EVA foam was attached on the bottom electrode to improve the uniformity of the applied pressures.

The detailed microscopic structural and morphological characterizations were carried out with HITACHI SU8020 field‐emission scanning electron microscope (FESEM) and a FEI Tecnai F20 high‐resolution transmission electron microscope (HRTEM) equipped with a nanoprobe energy‐dispersive (EDS) X‐ray spectroscope. XRD spectra were obtained with an X‐ray diffractometer (X'pert^3^ Powder). The solar cell was irradiated using a solar simulator (Model 94023A, Newport) with an AM 1.5 spectrum distribution calibrated against a NREL reference cell to accurately simulate full‐sun intensity (100 mW cm^−2^). The *J*–*V* characteristics of the device were recorded by an electrochemical workstation (CHI660E).

## Supporting information

As a service to our authors and readers, this journal provides supporting information supplied by the authors. Such materials are peer reviewed and may be re‐organized for online delivery, but are not copy‐edited or typeset. Technical support issues arising from supporting information (other than missing files) should be addressed to the authors.

Supplementary
