# Supplementary material for: Piezo‐Phototronic Effect Enhanced Flexible Solar Cells Based on n‐ZnO/p‐SnS Core–Shell Nanowire Array
Source: Adv Sci (Weinh). 2016 Jul 7;4(1):1600185. doi: 10.1002/advs.201600185 (PMC5238743; doi:10.1002/advs.201600185)
Supplement: Supplementary file 1 — Supplementary [file ADVS-4-0-s001.pdf]

Copyright WILEY-VCH Verlag GmbH & Co. KGaA, 69469 Weinheim, Germany, 2016.

ADVANCED SCIENCE
Open Access

Supporting Information

for *Adv. Sci.*, DOI: 10.1002/advs.201600185

Piezo-Phototronic Effect Enhanced Flexible Solar Cells Based on n-ZnO/p-SnS Core–Shell Nanowire Array

*Laipan Zhu, Longfei Wang, Fei Xue, Libo Chen, Jianqiang Fu, Xiaolong Feng, Tianfeng Li, and Zhong Lin Wang**

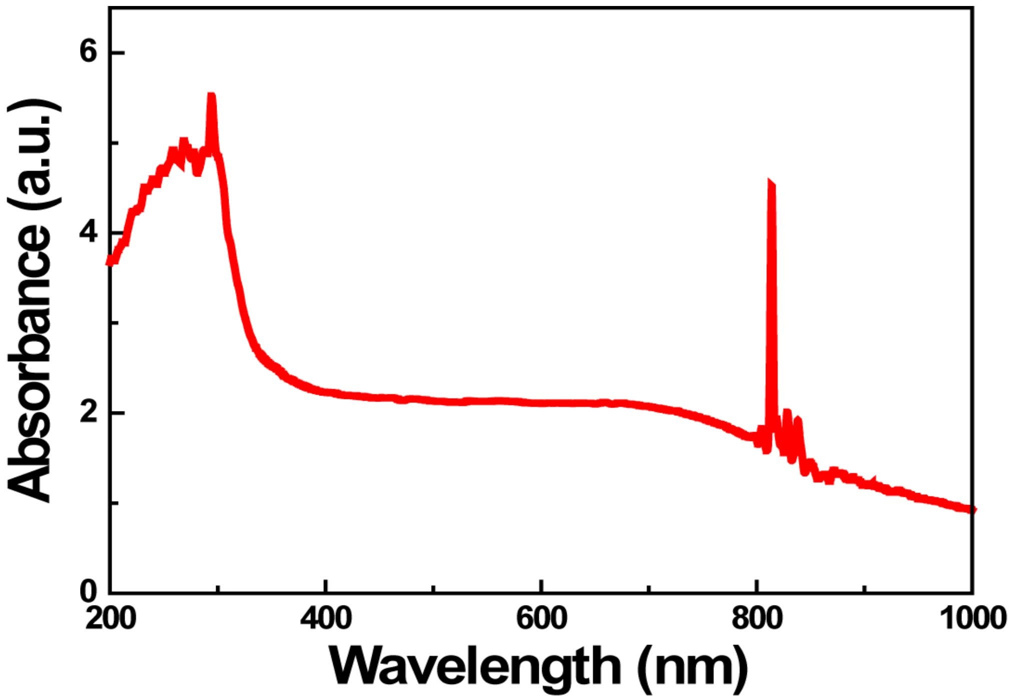

Figure S1. Absorption spectrum of the as-synthesized ZnO/SnS core-shell nanowire array.

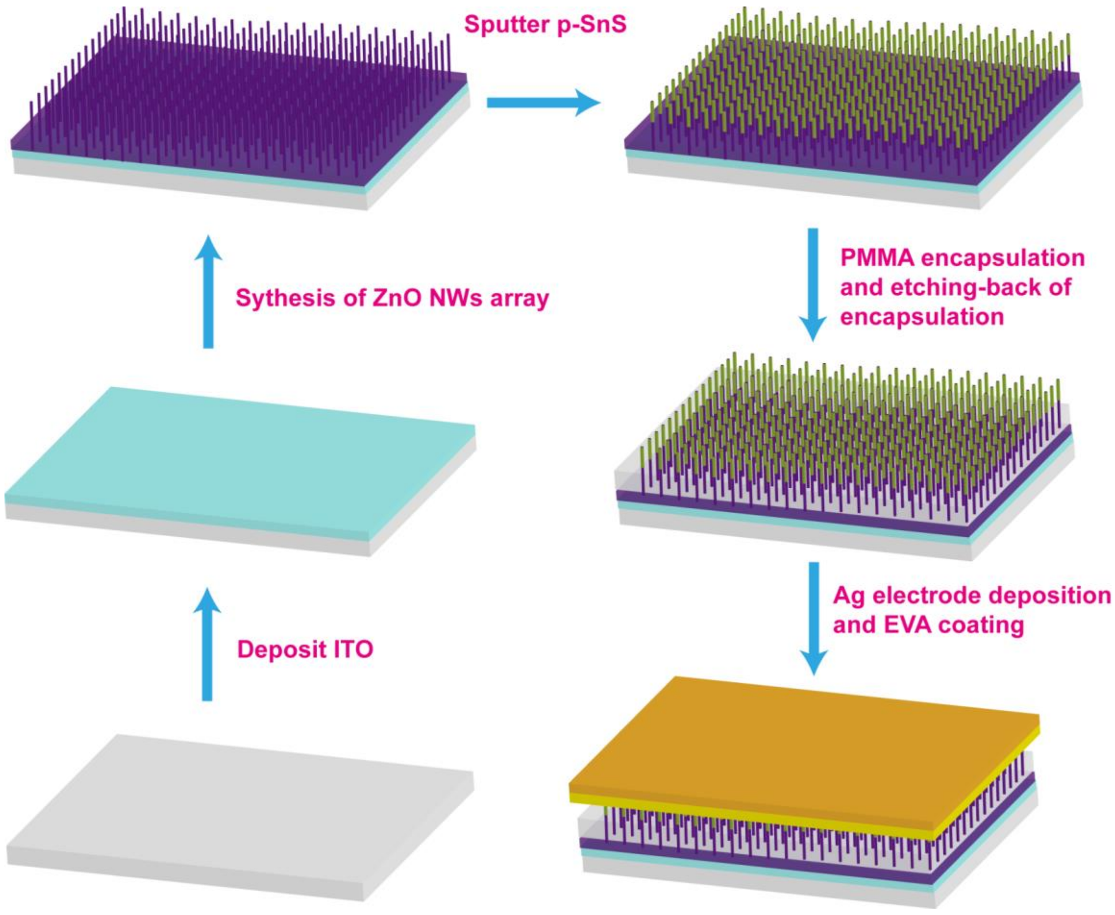

Figure S2. Schematic image of the device fabrication process.

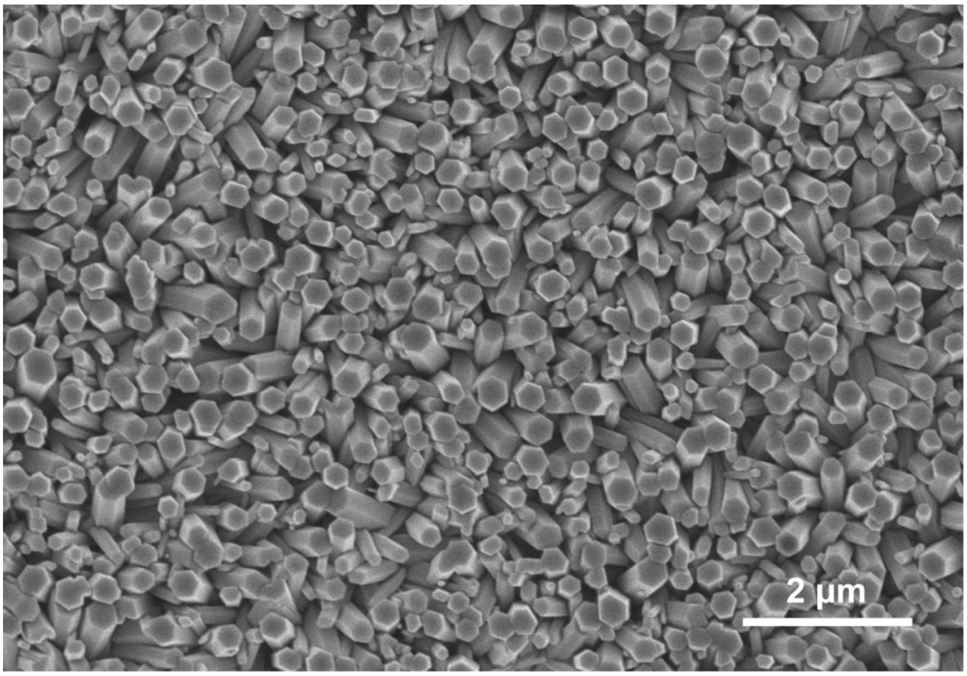

Figure S3. Top view of the SEM image of the as-synthesized ZnO nanowire array grown by hydrothermal process.

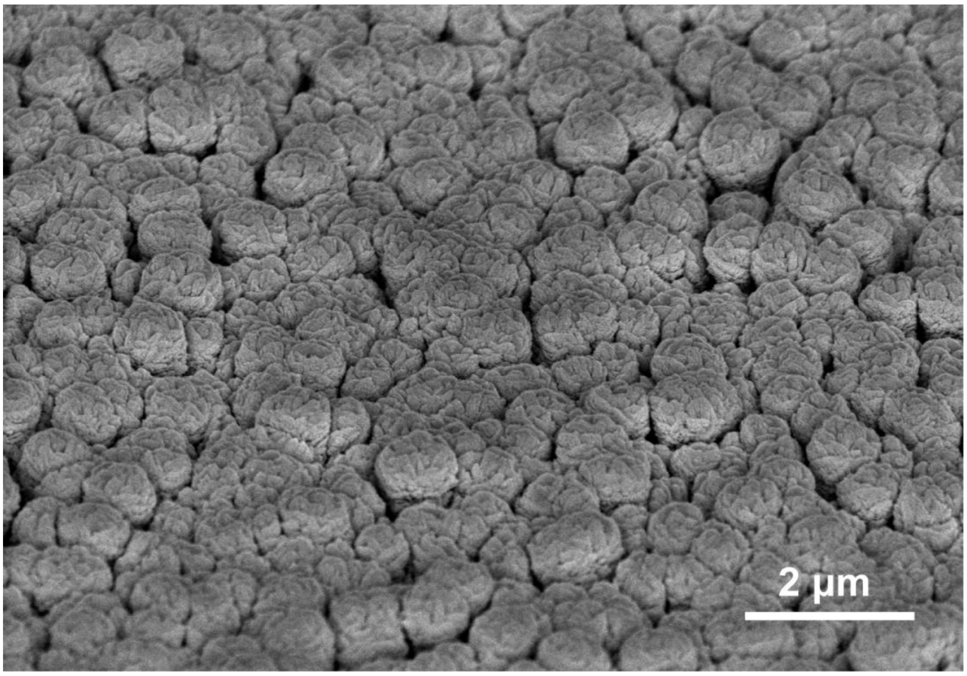

Figure S4. Top view of the SEM image of the as-synthesized ZnO/SnS core-shell nanowire array.
